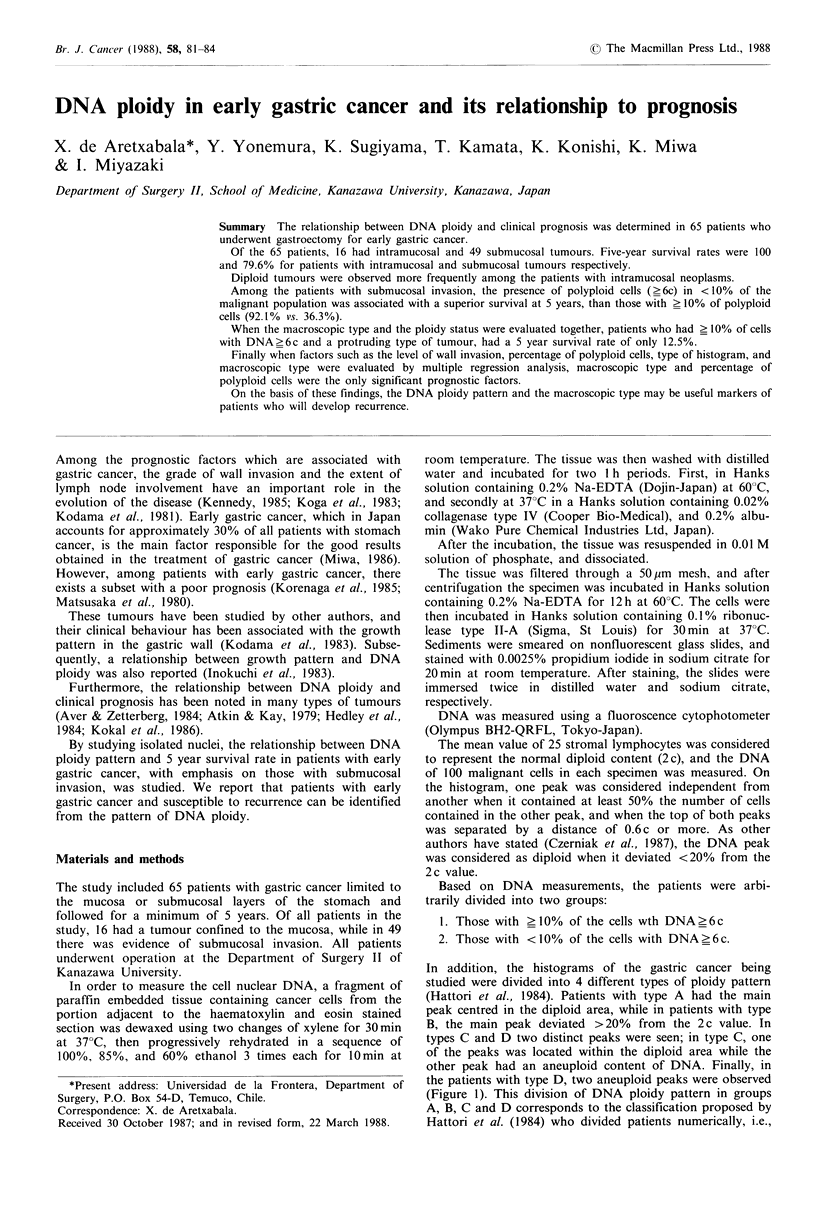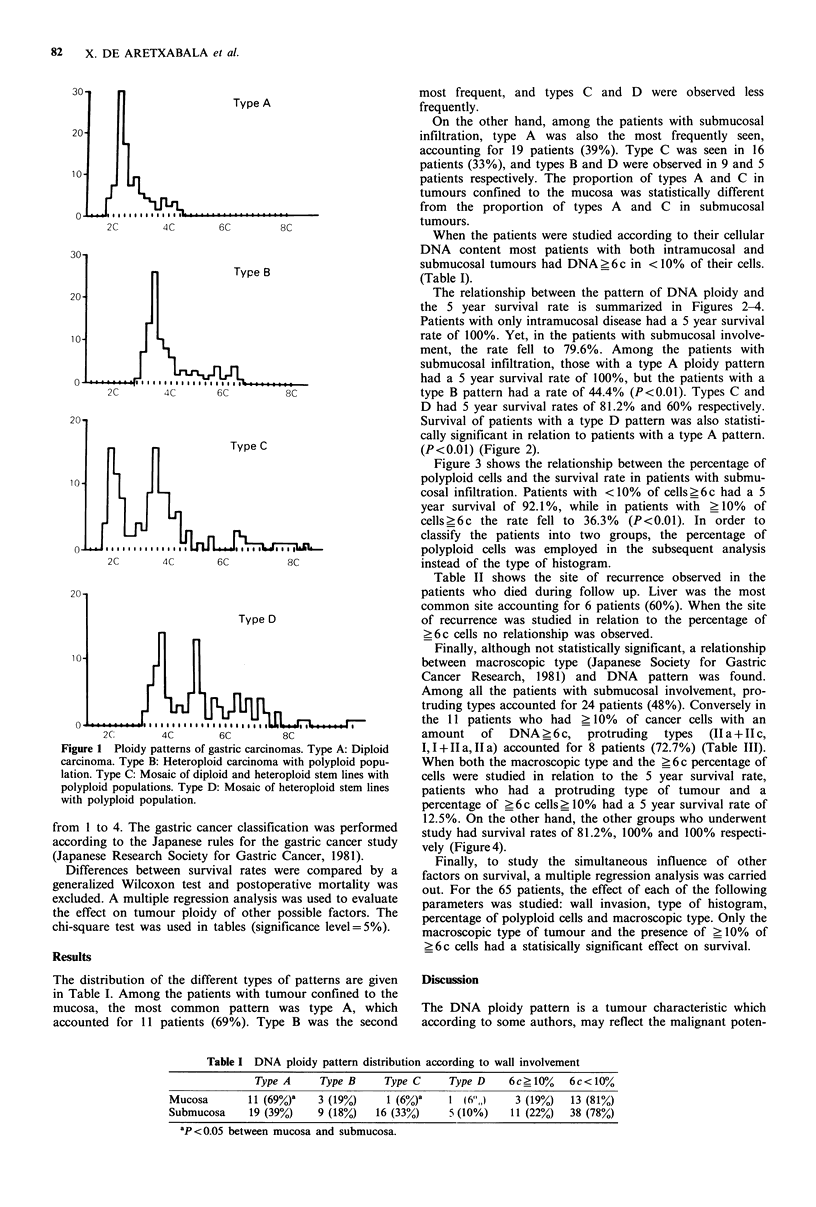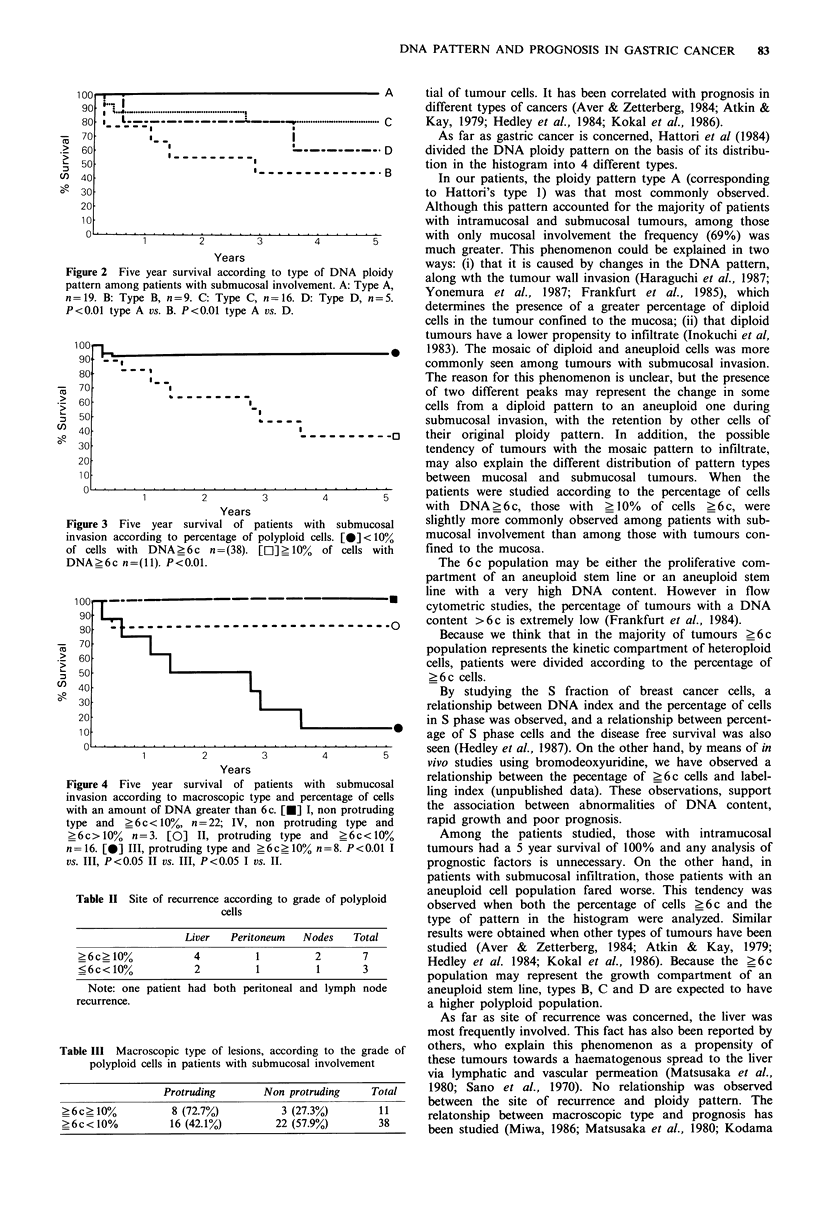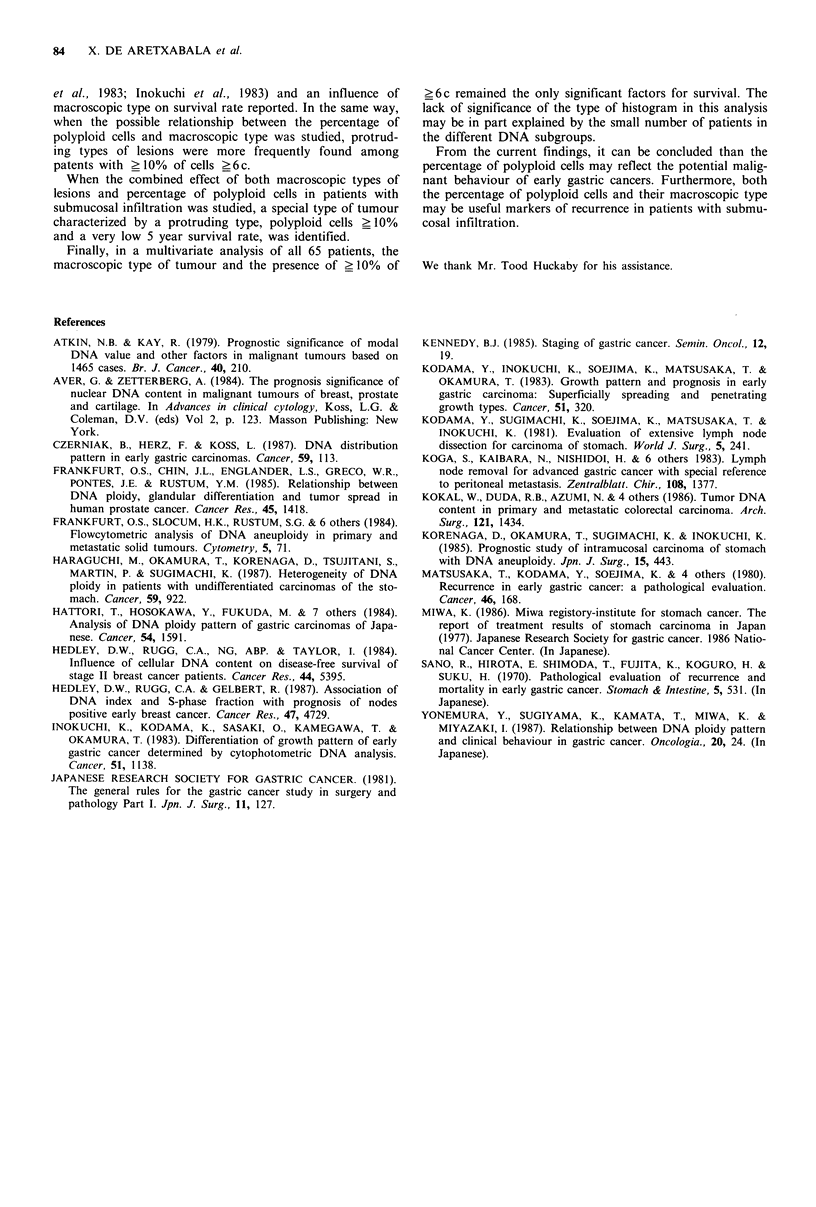# DNA ploidy in early gastric cancer and its relationship to prognosis.

**DOI:** 10.1038/bjc.1988.167

**Published:** 1988-07

**Authors:** X. de Aretxabala, Y. Yonemura, K. Sugiyama, T. Kamata, K. Konishi, K. Miwa, I. Miyazaki

**Affiliations:** Department of Surgery II, School of Medicine, Kanazawa University, Japan.

## Abstract

The relationship between DNA ploidy and clinical prognosis was determined in 65 patients who underwent gastroectomy for early gastric cancer. Of the 65 patients, 16 had intramucosal and 49 submucosal tumours. Five-year survival rates were 100 and 79.6% for patients with intramucosal and submucosal tumours respectively. Diploid tumours were observed more frequently among the patients with intramucosal neoplasms. Among the patients with submucosal invasion, the presence of polyploid cells (greater than or equal to 6c) in less than 10% of the malignant population was associated with a superior survival at 5 years, than those with greater than or equal to 10% of polyploid cells (92.1% vs. 36.3%). When the macroscopic type and the ploidy status were evaluated together, patients who had greater than or equal to 10% of cells with DNA greater than or equal to 6 c and a protruding type of tumour, had a 5 year survival rate of only 12.5%. Finally when factors such as the level of wall invasion, percentage of polyploid cells, type of histogram, and macroscopic type were evaluated by multiple regression analysis, macroscopic type and percentage of polyploid cells were the only significant prognostic factors. On the basis of these findings, the DNA ploidy pattern and the macroscopic type may be useful markers of patients who will develop recurrence.


*Br. J. Cancer* (1988), **58**, 81–84 © The Macmillan Press Ltd., 1988

### DNA ploidy in early gastric cancer and its relationship to prognosis

X. de Aretxabala*, Y. Yonemura, K. Sugiyama, T. Kamata, K. Konishi, K. Miwa & I. Miyazaki

*Department of Surgery II, School of Medicine, Kanazawa University, Kanazawa, Japan*

**Summary** The relationship between DNA ploidy and clinical prognosis was determined in 65 patients who underwent gastroectomy for early gastric cancer.

Of the 65 patients, 16 had intramucosal and 49 submucosal tumours. Five-year survival rates were 100 and 79.6% for patients with intramucosal and submucosal tumours respectively.

Diploid tumours were observed more frequently among the patients with intramucosal neoplasms.

Among the patients with submucosal invasion, the presence of polyploid cells (≧6c) in <10% of the malignant population was associated with a superior survival at 5 years, than those with ≧10% of polyploid cells (92.1% *vs.* 36.3%).

When the macroscopic type and the ploidy status were evaluated together, patients who had ≧10% of cells with DNA≧6c and a protruding type of tumour, had a 5 year survival rate of only 12.5%.

Finally when factors such as the level of wall invasion, percentage of polyploid cells, type of histogram, and macroscopic type were evaluated by multiple regression analysis, macroscopic type and percentage of polyploid cells were the only significant prognostic factors.

On the basis of these findings, the DNA ploidy pattern and the macroscopic type may be useful markers of patients who will develop recurrence.

Among the prognostic factors which are associated with gastric cancer, the grade of wall invasion and the extent of lymph node involvement have an important role in the evolution of the disease (Kennedy, 1985; Koga *et al.*, 1983; Kodama *et al.*, 1981). Early gastric cancer, which in Japan accounts for approximately 30% of all patients with stomach cancer, is the main factor responsible for the good results obtained in the treatment of gastric cancer (Miwa, 1986). However, among patients with early gastric cancer, there exists a subset with a poor prognosis (Korenaga *et al.*, 1985; Matsusaka *et al.*, 1980).

These tumours have been studied by other authors, and their clinical behaviour has been associated with the growth pattern in the gastric wall (Kodama *et al.*, 1983). Subsequently, a relationship between growth pattern and DNA ploidy was also reported (Inokuchi *et al.*, 1983).

Furthermore, the relationship between DNA ploidy and clinical prognosis has been noted in many types of tumours (Aver & Zetterberg, 1984; Atkin & Kay, 1979; Hedley *et al.*, 1984; Kokal *et al.*, 1986).

By studying isolated nuclei, the relationship between DNA ploidy pattern and 5 year survival rate in patients with early gastric cancer, with emphasis on those with submucosal invasion, was studied. We report that patients with early gastric cancer and susceptible to recurrence can be identified from the pattern of DNA ploidy.

### Materials and methods

The study included 65 patients with gastric cancer limited to the mucosa or submucosal layers of the stomach and followed for a minimum of 5 years. Of all patients in the study, 16 had a tumour confined to the mucosa, while in 49 there was evidence of submucosal invasion. All patients underwent operation at the Department of Surgery II of Kanazawa University.

In order to measure the cell nuclear DNA, a fragment of paraffin embedded tissue containing cancer cells from the portion adjacent to the haematoxylin and eosin stained section was dewaxed using two changes of xylene for 30 min at 37°C, then progressively rehydrated in a sequence of 100%, 85%, and 60% ethanol 3 times each for 10 min at room temperature. The tissue was then washed with distilled water and incubated for two 1 h periods. First, in Hanks solution containing 0.2% Na-EDTA (Dojin-Japan) at 60°C, and secondly at 37°C in a Hanks solution containing 0.02% collagenase type IV (Cooper Bio-Medical), and 0.2% albumin (Wako Pure Chemical Industries Ltd, Japan).

After the incubation, the tissue was resuspended in 0.01 M solution of phosphate, and dissociated.

The tissue was filtered through a 50 $\mu$m mesh, and after centrifugation the specimen was incubated in Hanks solution containing 0.2% Na-EDTA for 12 h at 60°C. The cells were then incubated in Hanks solution containing 0.1% ribonuclease type II-A (Sigma, St Louis) for 30 min at 37°C. Sediments were smeared on nonfluorescent glass slides, and stained with 0.0025% propidium iodide in sodium citrate for 20 min at room temperature. After staining, the slides were immersed twice in distilled water and sodium citrate, respectively.

DNA was measured using a fluoroscence cytophotometer (Olympus BH2-QRFL, Tokyo-Japan).

The mean value of 25 stromal lymphocytes was considered to represent the normal diploid content (2 c), and the DNA of 100 malignant cells in each specimen was measured. On the histogram, one peak was considered independent from another when it contained at least 50% the number of cells contained in the other peak, and when the top of both peaks was separated by a distance of 0.6 c or more. As other authors have stated (Czerniak *et al.*, 1987), the DNA peak was considered as diploid when it deviated <20% from the 2 c value.

Based on DNA measurements, the patients were arbitrarily divided into two groups:

1. Those with ≧10% of the cells wth DNA≧6 c
2. Those with <10% of the cells with DNA≧6 c.

In addition, the histograms of the gastric cancer being studied were divided into 4 different types of ploidy pattern (Hattori *et al.*, 1984). Patients with type A had the main peak centred in the diploid area, while in patients with type B, the main peak deviated >20% from the 2 c value. In types C and D two distinct peaks were seen; in type C, one of the peaks was located within the diploid area while the other peak had an aneuploid content of DNA. Finally, in the patients with type D, two aneuploid peaks were observed (Figure 1). This division of DNA ploidy pattern in groups A, B, C and D corresponds to the classification proposed by Hattori *et al.* (1984) who divided patients numerically, i.e.,

*Present address: Universidad de la Frontera, Department of Surgery, P.O. Box 54-D, Temuco, Chile.
Correspondence: X. de Aretxabala.
Received 30 October 1987; and in revised form, 22 March 1988.

**Figure 1** Ploidy patterns of gastric carcinomas. Type A: Diploid carcinoma. Type B: Heteroploid carcinoma with polyploid population. Type C: Mosaic of diploid and heteroploid stem lines with polyploid populations. Type D: Mosaic of heteroploid stem lines with polyploid population.

from 1 to 4. The gastric cancer classification was performed according to the Japanese rules for the gastric cancer study (Japanese Research Society for Gastric Cancer, 1981).

Differences between survival rates were compared by a generalized Wilcoxon test and postoperative mortality was excluded. A multiple regression analysis was used to evaluate the effect on tumour ploidy of other possible factors. The chi-square test was used in tables (significance level = 5%).

### Results

The distribution of the different types of patterns are given in Table I. Among the patients with tumour confined to the mucosa, the most common pattern was type A, which accounted for 11 patients (69%). Type B was the second most frequent, and types C and D were observed less frequently.

On the other hand, among the patients with submucosal infiltration, type A was also the most frequently seen, accounting for 19 patients (39%). Type C was seen in 16 patients (33%), and types B and D were observed in 9 and 5 patients respectively. The proportion of types A and C in tumours confined to the mucosa was statistically different from the proportion of types A and C in submucosal tumours.

When the patients were studied according to their cellular DNA content most patients with both intramucosal and submucosal tumours had DNA $\geqq 6$c in $< 10\%$ of their cells. (Table I).

The relationship between the pattern of DNA ploidy and the 5 year survival rate is summarized in Figures 2–4. Patients with only intramucosal disease had a 5 year survival rate of 100%. Yet, in the patients with submucosal involvement, the rate fell to 79.6%. Among the patients with submucosal infiltration, those with a type A ploidy pattern had a 5 year survival rate of 100%, but the patients with a type B pattern had a rate of 44.4% ($P < 0.01$). Types C and D had 5 year survival rates of 81.2% and 60% respectively. Survival of patients with a type D pattern was also statistically significant in relation to patients with a type A pattern. ($P < 0.01$) (Figure 2).

Figure 3 shows the relationship between the percentage of polyploid cells and the survival rate in patients with submucosal infiltration. Patients with $< 10\%$ of cells $\geqq 6$c had a 5 year survival of 92.1%, while in patients with $\geqq 10\%$ of cells $\geqq 6$c the rate fell to 36.3% ($P < 0.01$). In order to classify the patients into two groups, the percentage of polyploid cells was employed in the subsequent analysis instead of the type of histogram.

Table II shows the site of recurrence observed in the patients who died during follow up. Liver was the most common site accounting for 6 patients (60%). When the site of recurrence was studied in relation to the percentage of $\geqq 6$c cells no relationship was observed.

Finally, although not statistically significant, a relationship between macroscopic type (Japanese Society for Gastric Cancer Research, 1981) and DNA pattern was found. Among all the patients with submucosal involvement, protruding types accounted for 24 patients (48%). Conversely in the 11 patients who had $\geqq 10\%$ of cancer cells with an amount of DNA $\geqq 6$c, protruding types (IIa + IIc, I, I + IIa, IIa) accounted for 8 patients (72.7%) (Table III). When both the macroscopic type and the $\geqq 6$c percentage of cells were studied in relation to the 5 year survival rate, patients who had a protruding type of tumour and a percentage of $\geqq 6$c cells $\geqq 10\%$ had a 5 year survival rate of 12.5%. On the other hand, the other groups who underwent study had survival rates of 81.2%, 100% and 100% respectively (Figure 4).

Finally, to study the simultaneous influence of other factors on survival, a multiple regression analysis was carried out. For the 65 patients, the effect of each of the following parameters was studied: wall invasion, type of histogram, percentage of polyploid cells and macroscopic type. Only the macroscopic type of tumour and the presence of $\geqq 10\%$ of $\geqq 6$c cells had a statisically significant effect on survival.

### Discussion

The DNA ploidy pattern is a tumour characteristic which according to some authors, may reflect the malignant poten-

**Table I** DNA ploidy pattern distribution according to wall involvement

| | *Type A* | *Type B* | *Type C* | *Type D* | $6c \geqq 10\%$ | $6c < 10\%$ |
|---|---|---|---|---|---|---|
| Mucosa | 11 (69%)[a] | 3 (19%) | 1 (6%)[a] | 1 (6%) | 3 (19%) | 13 (81%) |
| Submucosa | 19 (39%) | 9 (18%) | 16 (33%) | 5 (10%) | 11 (22%) | 38 (78%) |

[a]$P < 0.05$ between mucosa and submucosa.

**Figure 2** Five year survival according to type of DNA ploidy pattern among patients with submucosal involvement. A: Type A, $n=19$. B: Type B, $n=9$. C: Type C, $n=16$. D: Type D, $n=5$. $P<0.01$ type A *vs.* B. $P<0.01$ type A *vs.* D.

**Figure 3** Five year survival of patients with submucosal invasion according to percentage of polyploid cells. [●] < 10% of cells with DNA≧6c $n=(38)$. [□] ≧ 10% of cells with DNA≧6c $n=(11)$. $P<0.01$.

**Figure 4** Five year survival of patients with submucosal invasion according to macroscopic type and percentage of cells with an amount of DNA greater than 6c. [■] I, non protruding type and ≧6c<10%, $n=22$; IV, non protruding type and ≧6c>10% $n=3$. [O] II, protruding type and ≧6c<10% $n=16$. [●] III, protruding type and ≧6c≧10% $n=8$. $P<0.01$ I *vs.* III, $P<0.05$ II *vs.* III, $P<0.05$ I *vs.* II.

**Table II** Site of recurrence according to grade of polyploid cells

| | *Liver* | *Peritoneum* | *Nodes* | *Total* |
|---|---|---|---|---|
| ≧6c≧10% | 4 | 1 | 2 | 7 |
| ≦6c<10% | 2 | 1 | 1 | 3 |

Note: one patient had both peritoneal and lymph node recurrence.

**Table III** Macroscopic type of lesions, according to the grade of polyploid cells in patients with submucosal involvement

| | *Protruding* | *Non protruding* | *Total* |
|---|---|---|---|
| ≧6c≧10% | 8 (72.7%) | 3 (27.3%) | 11 |
| ≧6c<10% | 16 (42.1%) | 22 (57.9%) | 38 |

tial of tumour cells. It has been correlated with prognosis in different types of cancers (Aver & Zetterberg, 1984; Atkin & Kay, 1979; Hedley *et al.*, 1984; Kokal *et al.*, 1986).

As far as gastric cancer is concerned, Hattori *et al* (1984) divided the DNA ploidy pattern on the basis of its distribution in the histogram into 4 different types.

In our patients, the ploidy pattern type A (corresponding to Hattori's type 1) was that most commonly observed. Although this pattern accounted for the majority of patients with intramucosal and submucosal tumours, among those with only mucosal involvement the frequency (69%) was much greater. This phenomenon could be explained in two ways: (i) that it is caused by changes in the DNA pattern, along wth the tumour wall invasion (Haraguchi *et al.*, 1987; Yonemura *et al.*, 1987; Frankfurt *et al.*, 1985), which determines the presence of a greater percentage of diploid cells in the tumour confined to the mucosa; (ii) that diploid tumours have a lower propensity to infiltrate (Inokuchi *et al*, 1983). The mosaic of diploid and aneuploid cells was more commonly seen among tumours with submucosal invasion. The reason for this phenomenon is unclear, but the presence of two different peaks may represent the change in some cells from a diploid pattern to an aneuploid one during submucosal invasion, with the retention by other cells of their original ploidy pattern. In addition, the possible tendency of tumours with the mosaic pattern to infiltrate, may also explain the different distribution of pattern types between mucosal and submucosal tumours. When the patients were studied according to the percentage of cells with DNA≧6c, those with ≧10% of cells ≧6c, were slightly more commonly observed among patients with submucosal involvement than among those with tumours confined to the mucosa.

The 6c population may be either the proliferative compartment of an aneuploid stem line or an aneuploid stem line with a very high DNA content. However in flow cytometric studies, the percentage of tumours with a DNA content >6c is extremely low (Frankfurt *et al.*, 1984).

Because we think that in the majority of tumours ≧6c population represents the kinetic compartment of heteroploid cells, patients were divided according to the percentage of ≧6c cells.

By studying the S fraction of breast cancer cells, a relationship between DNA index and the percentage of cells in S phase was observed, and a relationship between percentage of S phase cells and the disease free survival was also seen (Hedley *et al.*, 1987). On the other hand, by means of *in vivo* studies using bromodeoxyuridine, we have observed a relationship between the pecentage of ≧6c cells and labelling index (unpublished data). These observations, support the association between abnormalities of DNA content, rapid growth and poor prognosis.

Among the patients studied, those with intramucosal tumours had a 5 year survival of 100% and any analysis of prognostic factors is unnecessary. On the other hand, in patients with submucosal infiltration, those patients with an aneuploid cell population fared worse. This tendency was observed when both the percentage of cells ≧6c and the type of pattern in the histogram were analyzed. Similar results were obtained when other types of tumours have been studied (Aver & Zetterberg, 1984; Atkin & Kay, 1979; Hedley *et al.* 1984; Kokal *et al.*, 1986). Because the ≧6c population may represent the growth compartment of an aneuploid stem line, types B, C and D are expected to have a higher polyploid population.

As far as site of recurrence was concerned, the liver was most frequently involved. This fact has also been reported by others, who explain this phenomenon as a propensity of these tumours towards a haematogenous spread to the liver via lymphatic and vascular permeation (Matsusaka *et al.*, 1980; Sano *et al.*, 1970). No relationship was observed between the site of recurrence and ploidy pattern. The relatonship between macroscopic type and prognosis has been studied (Miwa, 1986; Matsusaka *et al.*, 1980; Kodama

*et al.*, 1983; Inokuchi *et al.*, 1983) and an influence of macroscopic type on survival rate reported. In the same way, when the possible relationship between the percentage of polyploid cells and macroscopic type was studied, protruding types of lesions were more frequently found among patents with $\geqq 10\%$ of cells $\geqq 6c$.

When the combined effect of both macroscopic types of lesions and percentage of polyploid cells in patients with submucosal infiltration was studied, a special type of tumour characterized by a protruding type, polyploid cells $\geqq 10\%$ and a very low 5 year survival rate, was identified.

Finally, in a multivariate analysis of all 65 patients, the macroscopic type of tumour and the presence of $\geqq 10\%$ of $\geqq 6c$ remained the only significant factors for survival. The lack of significance of the type of histogram in this analysis may be in part explained by the small number of patients in the different DNA subgroups.

From the current findings, it can be concluded than the percentage of polyploid cells may reflect the potential malignant behaviour of early gastric cancers. Furthermore, both the percentage of polyploid cells and their macroscopic type may be useful markers of recurrence in patients with submucosal infiltration.

We thank Mr. Tood Huckaby for his assistance.

### References

ATKIN, N.B. & KAY, R. (1979). Prognostic significance of modal DNA value and other factors in malignant tumours based on 1465 cases. *Br. J. Cancer.*, **40**, 210.

AVER, G. & ZETTERBERG, A. (1984). The prognosis significance of nuclear DNA content in malignant tumours of breast, prostate and cartilage. In *Advances in clinical cytology*, Koss, L.G. & Coleman, D.V. (eds) Vol 2, p. 123. Masson Publishing: New York.

CZERNIAK, B., HERZ, F. & KOSS, L. (1987). DNA distribution pattern in early gastric carcinomas. *Cancer*, **59**, 113.

FRANKFURT, O.S., CHIN, J.L., ENGLANDER, L.S., GRECO, W.R., PONTES, J.E. & RUSTUM, Y.M. (1985). Relationship between DNA ploidy, glandular differentiation and tumor spread in human prostate cancer. *Cancer Res.*, **45**, 1418.

FRANKFURT, O.S., SLOCUM, H.K., RUSTUM, S.G. & 6 others (1984). Flowcytometric analysis of DNA aneuploidy in primary and metastatic solid tumours. *Cytometry*, **5**, 71.

HARAGUCHI, M., OKAMURA, T., KORENAGA, D., TSUJITANI, S., MARTIN, P. & SUGIMACHI, K. (1987). Heterogeneity of DNA ploidy in patients with undifferentiated carcinomas of the stomach. *Cancer*, **59**, 922.

HATTORI, T., HOSOKAWA, Y., FUKUDA, M. & 7 others (1984). Analysis of DNA ploidy pattern of gastric carcinomas of Japanese. *Cancer*, **54**, 1591.

HEDLEY, D.W., RUGG, C.A., NG, ABP. & TAYLOR, I. (1984). Influence of cellular DNA content on disease-free survival of stage II breast cancer patients. *Cancer Res.*, **44**, 5395.

HEDLEY, D.W., RUGG, C.A. & GELBERT, R. (1987). Association of DNA index and S-phase fraction with prognosis of nodes positive early breast cancer. *Cancer Res.*, **47**, 4729.

INOKUCHI, K., KODAMA, K., SASAKI, O., KAMEGAWA, T. & OKAMURA, T. (1983). Differentiation of growth pattern of early gastric cancer determined by cytophotometric DNA analysis. *Cancer*, **51**, 1138.

JAPANESE RESEARCH SOCIETY FOR GASTRIC CANCER. (1981). The general rules for the gastric cancer study in surgery and pathology Part I. *Jpn. J. Surg.*, **11**, 127.

KENNEDY, B.J. (1985). Staging of gastric cancer. *Semin. Oncol.*, **12**, 19.

KODAMA, Y., INOKUCHI, K., SOEJIMA, K., MATSUSAKA, T. & OKAMURA, T. (1983). Growth pattern and prognosis in early gastric carcinoma: Superficially spreading and penetrating growth types. *Cancer*, **51**, 320.

KODAMA, Y., SUGIMACHI, K., SOEJIMA, K., MATSUSAKA, T. & INOKUCHI, K. (1981). Evaluation of extensive lymph node dissection for carcinoma of stomach. *World J. Surg.*, **5**, 241.

KOGA, S., KAIBARA, N., NISHIDOI, H. & 6 others 1983). Lymph node removal for advanced gastric cancer with special reference to peritoneal metastasis. *Zentralblatt. Chir.*, **108**, 1377.

KOKAL, W., DUDA, R.B., AZUMI, N. & 4 others (1986). Tumor DNA content in primary and metastatic colorectal carcinoma. *Arch. Surg.*, **121**, 1434.

KORENAGA, D., OKAMURA, T., SUGIMACHI, K. & INOKUCHI, K. (1985). Prognostic study of intramucosal carcinoma of stomach with DNA aneuploidy. *Jpn. J. Surg.*, **15**, 443.

MATSUSAKA, T., KODAMA, Y., SOEJIMA, K. & 4 others (1980). Recurrence in early gastric cancer: a pathological evaluation. *Cancer*, **46**, 168.

MIWA, K. (1986). Miwa registory-institute for stomach cancer. The report of treatment results of stomach carcinoma in Japan (1977). Japanese Research Society for gastric cancer. 1986 National Cancer Center. (In Japanese).

SANO, R., HIROTA, E. SHIMODA, T., FUJITA, K., KOGURO, H. & SUKU, H. (1970). Pathological evaluation of recurrence and mortality in early gastric cancer. *Stomach & Intestine*, **5**, 531. (In Japanese).

YONEMURA, Y., SUGIYAMA, K., KAMATA, T., MIWA, K. & MIYAZAKI, I. (1987). Relationship between DNA ploidy pattern and clinical behaviour in gastric cancer. *Oncologia.*, **20**, 24. (In Japanese).